# A user centered evaluation framework for mobile health applications using Fuzzy AHP and TOPSIS: evidence from expert reviews and user experience data

**DOI:** 10.3389/fpubh.2026.1907690

**Published:** 2026-08-14

**Authors:** Xinrui Huang, Kaidi Ma

**Affiliations:** 1 School of Social Work, University of Pittsburgh, Pittsburgh, PA, United States; 2 AI Institute, Jiangsu XCMG State Key Laboratory Technology Co., Ltd., Xuzhou, Jiangsu, China

**Keywords:** clinical relevance, digital mental health, Fuzzy AHP, mobile health applications, multi-criteria decision making, privacy, TOPSIS, usability

## Abstract

**Background:**

The rapid growth of digital mental health applications has created a need for comprehensive evaluation approaches that simultaneously consider clinical effectiveness, user experience, and ethical requirements. Existing assessment methods often focus on limited aspects of application quality and may not adequately capture uncertainty in expert judgments.

**Objective:**

This study aimed to develop and apply a hybrid Fuzzy Analytic Hierarchy Process (Fuzzy AHP) and Technique for Order Preference by Similarity to Ideal Solution (TOPSIS) framework for the user-centered evaluation and ranking of digital mental health applications.

**Methods:**

A publicly available Mobile Health Apps Evaluation Dataset comprising 62 applications and 274 evaluation records was analyzed. Four main criteria and twelve subcriteria were established through expert consultation, including usability, engagement, clinical relevance, and ethics and privacy. An expert panel consisting of 18 specialists from public health, healthcare, digital health, health informatics, and user experience fields participated in the weighting process. Fuzzy AHP was employed to determine criteria weights under uncertainty, while TOPSIS was used to rank the selected applications. Sensitivity analysis was conducted to evaluate the robustness of the ranking outcomes.

**Results:**

Clinical relevance received the highest importance weight (0.352), followed by ethics and privacy (0.284), usability (0.211), and engagement (0.153). Among the subcriteria, clinical expert involvement (0.132), privacy policy availability (0.118), and guideline compliance (0.115) were identified as the most influential factors. The TOPSIS analysis ranked A1, A2, and A3 as the highest-performing applications, with closeness coefficients of 0.842, 0.816, and 0.791, respectively. Sensitivity analysis demonstrated strong ranking stability across alternative weighting scenarios, with Spearman correlation coefficients exceeding 0.90.

**Conclusion:**

The proposed hybrid Fuzzy AHP–TOPSIS framework provides a robust and transparent approach for evaluating digital mental health applications. The findings highlight the importance of clinical credibility, privacy protection, and evidence-based design in determining application quality. The framework offers practical guidance for developers, healthcare professionals, and policymakers seeking to identify and promote high-quality digital mental health technologies.

## 1 Introduction

Mental health disorders represent a major global public health challenge and continue to contribute substantially to disability, reduced quality of life, and socioeconomic burden (1, 2). The increasing prevalence of anxiety, depression, stress-related disorders, and other psychological conditions has intensified demand for accessible and scalable mental health services (3, 4). However, barriers such as limited healthcare resources, geographical constraints, stigma, and treatment costs continue to restrict access to conventional mental health care (5, 6).

The rapid advancement of digital health technologies has facilitated the emergence of digital mental health applications as complementary tools for mental health promotion, self-management, symptom monitoring, and psychological intervention (7, 8). Mobile applications offer several advantages, including continuous accessibility, personalized support, cost-effectiveness, and the potential to reach underserved populations (9, 10). Consequently, thousands of mental health applications are currently available through commercial app marketplaces, offering services ranging from mindfulness training and cognitive behavioral therapy to mood tracking and telepsychological support (11, 12).

Despite their increasing popularity, substantial concerns remain regarding the quality, effectiveness, safety, and transparency of digital mental health applications (13–15). Recent evidence indicates that many applications lack clinical validation, professional involvement, and adequate privacy protections, raising questions regarding their reliability and suitability for healthcare use (16, 17). Furthermore, application quality is inherently multidimensional and extends beyond usability considerations to include clinical relevance, user engagement, ethical responsibility, and privacy protection (18–20).

Evaluating digital mental health applications, therefore, presents a complex decision-making problem involving multiple, often conflicting criteria. Traditional evaluation approaches frequently rely on descriptive assessments, user ratings, or isolated quality indicators, which may fail to capture the multidimensional nature of application quality (21–23). Multi-criteria decision-making (MCDM) methods provide a structured approach for addressing such complexity by simultaneously considering multiple evaluation dimensions and stakeholder perspectives.

Among MCDM techniques, the Analytic Hierarchy Process (AHP) has been widely employed to determine the importance of criteria, whereas the Technique for Order Preference by Similarity to Ideal Solution (TOPSIS) has been extensively used for ranking alternatives (24). However, conventional AHP may be limited when expert judgments are uncertain and ambiguous. Fuzzy AHP addresses this limitation by incorporating fuzzy set theory into the weighting process, thereby providing a more realistic representation of human decision-making. The integration of Fuzzy AHP and TOPSIS offers a robust framework capable of deriving criteria weights under uncertainty and generating comprehensive rankings of competing alternatives (25).

Accordingly, this study proposes a hybrid Fuzzy AHP–TOPSIS framework for evaluating digital mental health applications from a user-centered perspective. By combining expert knowledge with application performance data, the study seeks to provide a transparent and systematic approach for assessing application quality and identifying high-performing digital mental health solutions.

### 1.1 Literature review

The evaluation of digital mental health applications has attracted increasing attention within the digital health and public health literature. Existing studies have examined application quality through diverse perspectives, including usability assessment, clinical effectiveness, privacy protection, user engagement, and regulatory compliance.

Recent systematic reviews have highlighted the rapid expansion of digital mental health technologies and the growing demand for evidence-based evaluation frameworks. Torous et al. (17) emphasized that although mental health applications continue to proliferate, substantial variability exists regarding clinical evidence, implementation quality, and healthcare integration. Similarly, Chowdhury et al. (25) reported that applications grounded in validated therapeutic approaches are more effective than those lacking clinical foundations.

Privacy and ethical concerns have also emerged as major evaluation dimensions. Lin et al. (16) identified significant inconsistencies in privacy policy quality and data protection practices among mental health applications. Likewise, Botes (11) reported that many applications fail to provide adequate transparency regarding data collection and sharing practices, potentially undermining user trust and long-term adoption.

Several studies have focused on usability and engagement as determinants of application acceptance and continued use. Valentine et al. (26) further reported that engagement mechanisms and persuasive design features contribute positively to adherence and intervention effectiveness. Nevertheless, usability and engagement alone are insufficient indicators of overall application quality because clinically ineffective or ethically problematic applications may still achieve high user satisfaction scores.

To address the multidimensional nature of digital health evaluation, researchers have increasingly adopted MCDM approaches. AHP, TOPSIS, VIKOR, DEMATEL, and PROMETHEE have been applied in healthcare technology assessment, telemedicine evaluation, and digital health prioritization. However, most existing studies focus on healthcare systems, medical devices, or telehealth services rather than digital mental health applications. Furthermore, many evaluations rely exclusively on either weighting methods or ranking techniques without integrating both approaches within a unified framework.

Recent MCDM studies have demonstrated that hybrid approaches provide superior analytical capabilities by combining the strengths of multiple decision-making methods. In particular, the integration of Fuzzy AHP and TOPSIS enables uncertainty-aware criteria weighting while maintaining a robust ranking mechanism. Despite these advantages, limited research has applied hybrid Fuzzy AHP–TOPSIS models to evaluate digital mental health applications using user-centered criteria that simultaneously encompass usability, engagement, clinical relevance, and ethics and privacy.

Several standardized frameworks have been developed to assess the quality of mobile health applications. The Mobile App Rating Scale (MARS) is among the most widely adopted instruments and evaluates applications according to engagement, functionality, aesthetics, and information quality (27). Although MARS provides a structured and reliable assessment process, it assigns equal importance to evaluation dimensions and does not account for uncertainty in expert judgments or provide an objective ranking of competing applications.

The Enlight Quality Assessment Tool extends evaluation by incorporating usability, therapeutic persuasiveness, user engagement, content quality, and privacy (28). Similarly, the American Psychiatric Association (APA) App Evaluation Framework emphasizes accessibility, privacy and security, clinical evidence, usability, and interoperability when selecting mental health applications (29). These frameworks provide valuable guidance for assessing application quality but rely primarily on qualitative or checklist-based assessments rather than quantitative decision-making methods.

Other assessment initiatives, including the Organization for the Review of Care and Health Applications (ORCHA) review methodology and the NHS Digital Assessment Questionnaire, evaluate applications according to clinical assurance, technical performance, regulatory compliance, and data governance. While these approaches facilitate quality certification, they do not estimate the relative importance of evaluation criteria or generate quantitative rankings that support evidence-based comparison among multiple applications.

To highlight the contribution of the proposed framework, Table 1 compares its main characteristics with widely used evaluation approaches.

**Table 1:** Comparison of existing mobile health evaluation frameworks and the proposed framework.

Framework	Main evaluation dimensions	Quantitative weighting	Application ranking	Handles uncertainty	Primary purpose
MARS	Engagement, functionality, aesthetics, information	No	No	No	Quality assessment
Enlight	Usability, engagement, therapeutic value, privacy	No	No	No	Quality assessment
APA App Evaluation Framework	Accessibility, privacy, evidence, usability	No	No	No	Clinical selection guidance
ORCHA Review	Clinical assurance, privacy, technical quality	No	No	No	Certification and appraisal
Proposed Fuzzy AHP–TOPSIS framework	Usability, engagement, clinical relevance, ethics, and privacy	Yes	Yes	Yes	Decision support and application ranking

Unlike previous frameworks, the proposed model combines qualitative assessment dimensions with quantitative multi-criteria decision-making. By integrating Fuzzy AHP and TOPSIS, the framework estimates the relative importance of evaluation criteria under uncertainty and subsequently ranks competing applications according to their overall performance. This integration provides a transparent and reproducible decision support approach that complements existing evaluation frameworks while addressing several of their methodological limitations.

### 1.2 Research gaps and objectives

Although digital mental health applications have been widely investigated, several important research gaps remain. First, existing evaluation studies frequently emphasize usability, engagement, or clinical effectiveness individually, while limited attention has been given to the simultaneous consideration of clinical relevance, ethics and privacy, usability, and engagement within a unified framework. Second, many assessment approaches rely on descriptive methods or user ratings, which may not adequately capture the complexity of multi-dimensional decision-making.

Third, uncertainty in expert judgments remains insufficiently addressed in current evaluations of digital mental health applications. Conventional weighting approaches often assume precise judgments despite the subjective nature of expert assessments. Fourth, relatively few studies have integrated advanced MCDM techniques to simultaneously derive criteria weights and rank digital mental health applications. Consequently, a gap remains in the development of comprehensive, uncertainty-aware evaluation frameworks that support evidence-based decision-making.

To address these gaps, this study proposes a hybrid Fuzzy AHP–TOPSIS framework for evaluating digital mental health applications. Specifically, the objectives of this study are:

1. To develop a user-centered evaluation framework for digital mental health applications based on usability, engagement, clinical relevance, and ethics and privacy criteria.
2. To determine the relative importance of evaluation criteria and subcriteria using the Fuzzy Analytic Hierarchy Process.
3. To rank digital mental health applications using the Technique for Order Preference by Similarity to Ideal Solution.
4. To evaluate the robustness of the ranking outcomes through sensitivity analysis.
5. To provide practical recommendations for developers, healthcare professionals, and policymakers regarding the evaluation and selection of high-quality digital mental health applications.

Unlike previous studies that primarily rely on existing evaluation checklists or single MCDM techniques, this study integrates uncertainty-aware expert weighting with standardized application evaluation data into a unified decision support framework. Rather than proposing another scoring system, the framework demonstrates how expert knowledge and user-experience indicators can be combined to produce transparent and reproducible rankings of digital mental health applications. This integration extends existing evaluation practice by providing a transferable methodological framework adaptable to different digital health domains. The framework is considered user-centered because it explicitly incorporates dimensions that determine user acceptance, usability, engagement, trust, and perceived safety while integrating expert judgments to estimate their relative importance.

## 2 Methodology

### 2.1 Study design

This study adopts a hybrid multi-criteria decision-making (MCDM) approach to evaluate mobile health applications from a user-centered perspective. The proposed framework integrates Fuzzy Analytic Hierarchy Process (Fuzzy AHP) and Technique for Order Preference by Similarity to Ideal Solution (TOPSIS) to address both subjective and objective aspects of application assessment. Fuzzy AHP is employed to determine the relative importance of evaluation criteria under uncertainty, while TOPSIS is used to rank mobile health applications according to their overall performance.

The study uses an open dataset of mobile health application evaluations comprising expert assessments of multiple health-related applications. The complete dataset is provided in the Supplementary material to ensure transparency and reproducibility of the research. Application-level evaluation records in the dataset serve as the basis for constructing the decision matrix for the TOPSIS analysis.

**Figure 1:**
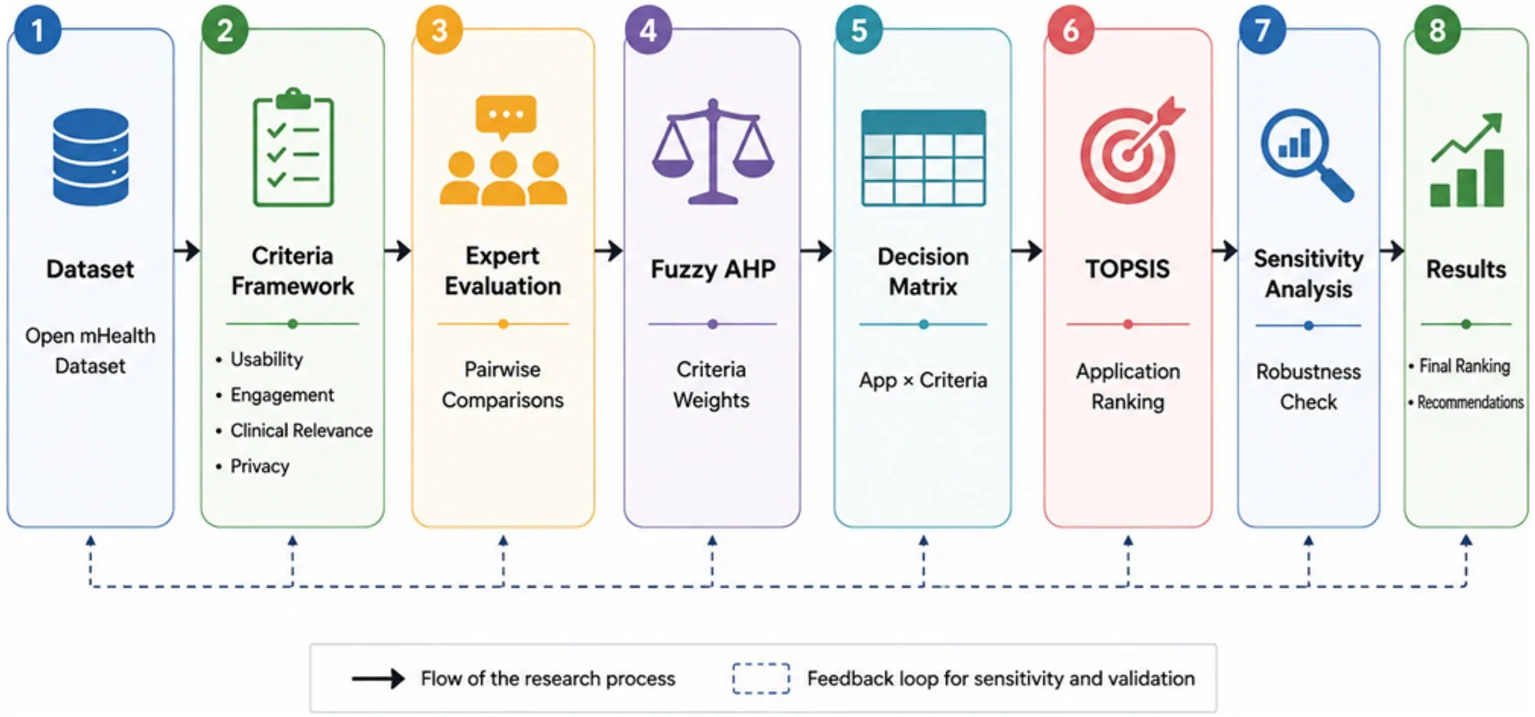
Research design of the hybrid Fuzzy AHP–TOPSIS framework for evaluating mobile health applications.

As illustrated in Figure 1, the research design consists of five sequential stages. First, an evaluation framework is established based on four major dimensions of mobile health application quality: usability, engagement, clinical relevance, and ethics and privacy. Second, expert judgments are collected to conduct pairwise comparisons among the evaluation criteria. Third, Fuzzy AHP is applied to calculate the relative weights of the criteria. Fourth, application performance data obtained from the open dataset are processed and transformed into a decision matrix. Finally, TOPSIS is employed to determine the relative closeness of each application to the ideal solution and to generate the final ranking.

The integration of Fuzzy AHP and TOPSIS provides a comprehensive evaluation framework that combines subjective expert knowledge with empirical application assessment data. This hybrid approach enables a systematic and transparent assessment of mobile health applications and supports evidence-based decision-making for healthcare professionals, application developers, and public health stakeholders.

### 2.2 Data source and application selection

Application performance data were obtained from the Mobile Health Apps Evaluation Dataset, an openly accessible dataset available through Kaggle. The dataset contains expert evaluations of mobile health applications across multiple dimensions, including usability, engagement, clinical relevance, privacy, and safety. A total of 62 mobile health applications and 274 evaluation records are included.

The dataset was selected because it provides standardized assessment measures that are consistent with the objectives of the present study. Its comprehensive coverage of application characteristics facilitates the implementation of a multi-criteria decision-making framework.

**Figure 2:**
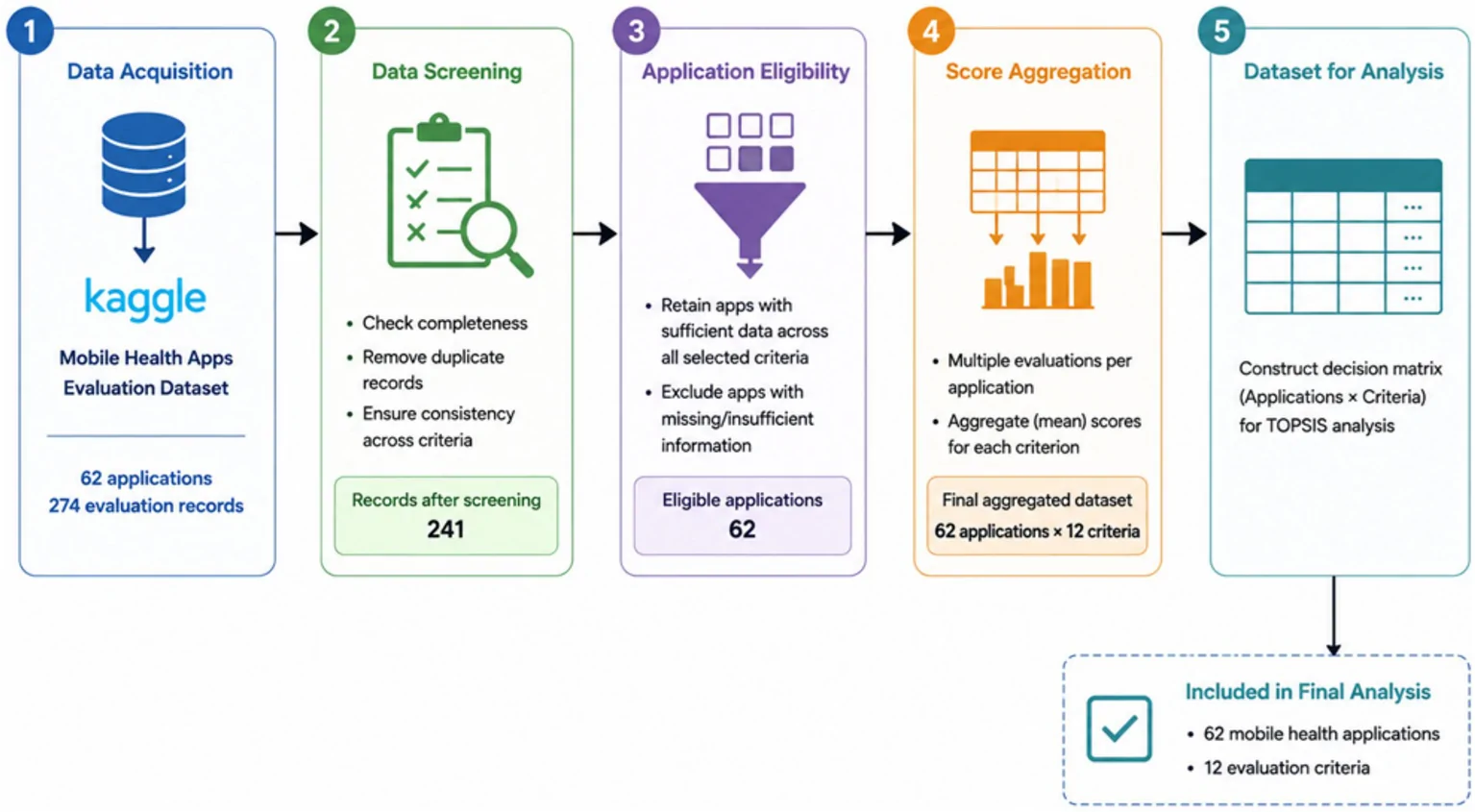
Dataset screening, preprocessing, and application selection process for constructing the TOPSIS decision matrix.

As illustrated in Figure 2, the application selection process involved data acquisition, screening, preprocessing, and aggregation. First, the complete dataset was obtained from the public repository. Second, records were examined for completeness and consistency. Third, applications with sufficient information across the selected evaluation criteria were retained for analysis. Finally, where multiple evaluations existed for the same application, criterion scores were aggregated to generate a single representative value for each application.

The resulting dataset was used to construct the decision matrix for the TOPSIS analysis, while expert judgments were employed separately within the Fuzzy AHP procedure to determine criterion weights. The complete dataset has been provided as Supplementary material to support transparency and reproducibility.

### 2.3 Development of the evaluation criteria framework

The evaluation criteria framework was developed to provide a comprehensive and theoretically grounded assessment of mobile health applications from a user-centered perspective. Rather than relying on a single quality dimension, the framework integrates technical, clinical, behavioral, and ethical aspects that jointly influence the effectiveness, acceptability, and long-term use of digital mental health applications. The selection of the evaluation criteria was informed by three complementary sources: established digital health evaluation frameworks, empirical evidence from the mobile health literature, and the characteristics of the Mobile Health Apps Evaluation Dataset used in this study.

Several established frameworks have demonstrated that the quality of mobile health applications cannot be adequately evaluated using usability or functionality alone. The Mobile App Rating Scale (MARS) emphasizes engagement, functionality, aesthetics, and information quality as essential indicators of application quality (27). Similarly, the Enlight Quality Assessment Tool extends evaluation beyond usability by incorporating therapeutic persuasiveness, user engagement, content quality, and privacy considerations (28). More recently, the American Psychiatric Association (APA) App Evaluation Framework has highlighted the importance of clinical credibility, evidence-based practice, transparency, and data security when selecting digital mental health applications (30). Collectively, these frameworks indicate that effective evaluation requires consideration of multiple interrelated dimensions rather than isolated performance indicators.

Based on this evidence, four primary evaluation criteria were selected for the proposed framework: usability, engagement, clinical relevance, and ethics and privacy. These dimensions capture both user-related experiences and healthcare requirements, while remaining compatible with the variables available in the public evaluation dataset.

*Usability* was selected because it represents one of the strongest determinants of technology acceptance and continued application use. According to the international standard ISO 9241-210, user-centered design requires systems to be effective, efficient, and satisfying for their intended users (31). Panduwiyasa et al. (32) showed that applications with intuitive interfaces, clear navigation, and reduced cognitive burden achieve higher adoption and retention rates among users experiencing mental health conditions. Therefore, usability was represented through the subcriteria of ease of use, learnability, and user confidence. These indicators reflect both the accessibility of application functions and users’ ability to interact successfully with the digital intervention (32).

*Engagement* was included because sustained interaction is essential for achieving meaningful health outcomes. Unlike conventional software, digital mental health applications depend on repeated and long-term user participation to deliver therapeutic benefits. Previous studies have reported that engagement features such as interactive feedback, reminders, personalization, and motivational design positively influence adherence and intervention effectiveness (28, 33). Consequently, the proposed framework evaluates engagement using interaction quality, intention to use, and user engagement mechanisms. These subcriteria assess the extent to which applications encourage continuous participation and sustain user interest throughout the intervention.

*Clinical relevance* was identified as the most important healthcare-oriented dimension, as digital mental health applications increasingly serve as adjuncts to clinical care rather than mere wellness tools. Numerous studies have demonstrated that applications supported by healthcare professionals and evidence based therapeutic approaches produce better clinical outcomes than applications lacking scientific validation (34). Furthermore, regulatory organizations increasingly recommend that digital health technologies demonstrate clinical credibility, guideline compliance, and integration with healthcare services before widespread adoption. Therefore, clinical relevance was represented by clinical expert involvement, guideline compliance, and healthcare integration. These subcriteria evaluate whether applications are supported by professional expertise and aligned with established clinical practice.

*Ethics and privacy* were incorporated because digital mental health applications routinely collect sensitive personal information, including psychological symptoms, behavioral patterns, and health records. Inadequate privacy protection may reduce user trust and discourage adoption despite otherwise favorable application performance. Previous investigations have identified substantial variation in privacy policy transparency, data sharing practices, and security safeguards among commercially available mental health applications (35). International organizations, including the World Health Organization, have also emphasized that responsible digital health implementation requires robust governance, data protection, and ethical accountability (29). Consequently, ethics and privacy were evaluated based on the availability of privacy policies, data-sharing transparency, and safety alerts.

The proposed hierarchical framework therefore integrates dimensions representing user experience, behavioral engagement, healthcare quality, and ethical responsibility within a single evaluation structure. Unlike existing qualitative assessment tools that provide descriptive ratings, the present framework enables quantitative weighting of these criteria through Fuzzy AHP while simultaneously ranking competing applications using TOPSIS. This integration allows uncertainty in expert judgments to be explicitly considered while maintaining a transparent and reproducible decision-making process. The resulting framework is therefore intended to support developers, healthcare professionals, policymakers, and researchers in identifying high-quality digital mental health applications based on multiple evidence-informed criteria rather than individual quality indicators.

**Figure 3:**
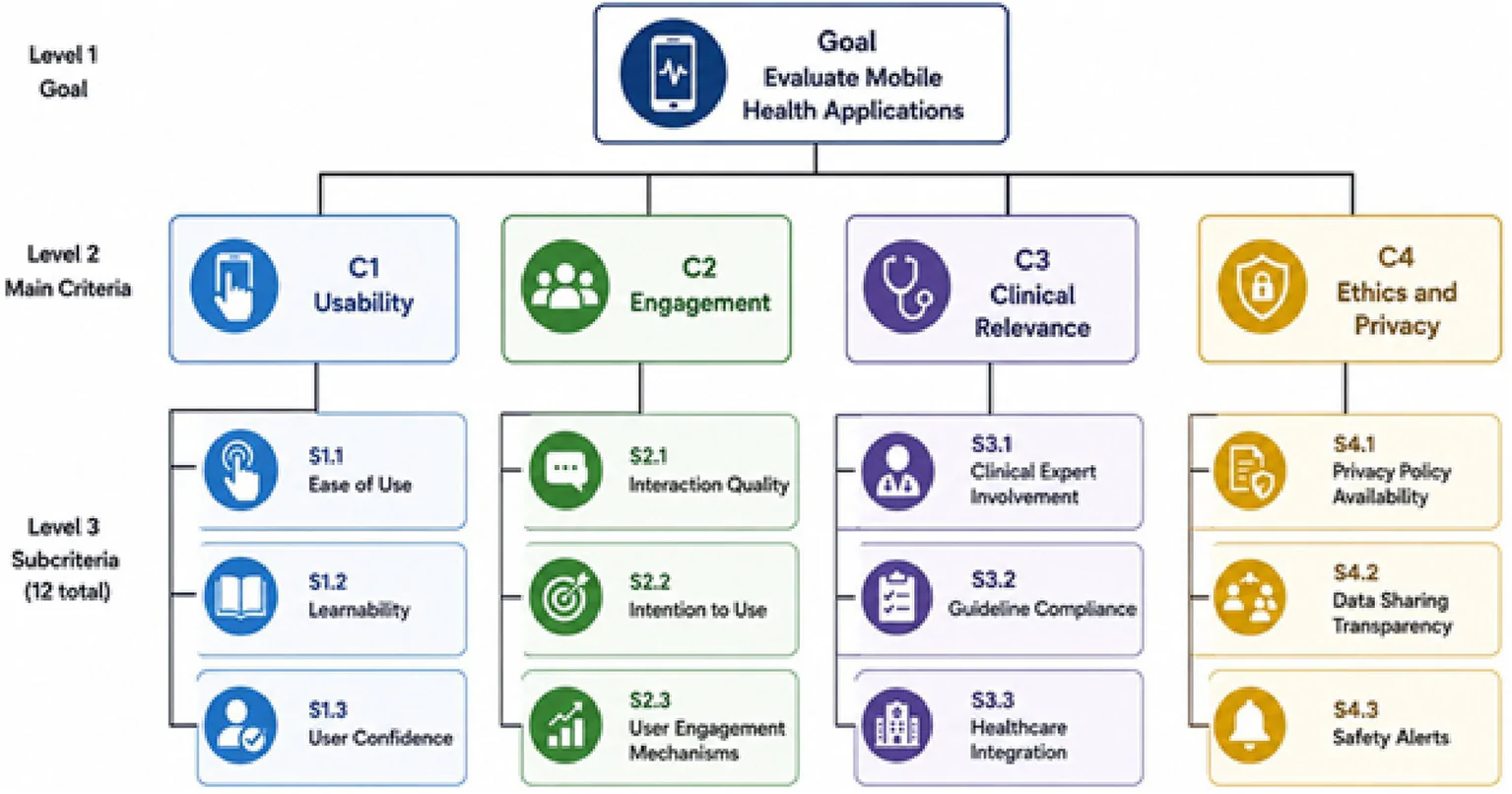
Hierarchical evaluation framework comprising the overall evaluation goal, four main evaluation criteria (usability, engagement, clinical relevance, and ethics and privacy), and twelve corresponding subcriteria for assessing mobile health applications.

As shown in Figure 3, the proposed hierarchical framework consists of one overall goal, four main criteria, and twelve subcriteria. Usability was assessed through ease of use, learnability, and user confidence. Engagement was evaluated using interaction quality, intention to use, and user engagement mechanisms. Clinical relevance was measured through clinical expert involvement, guideline compliance, and healthcare integration. Ethics and privacy were assessed based on the availability of privacy policies, data-sharing transparency, and safety alerts.

### 2.4 Expert panel and data collection

Expert judgments were collected to determine the relative importance of the evaluation criteria within the proposed Fuzzy AHP framework. A purposive sampling strategy was adopted to recruit individuals with demonstrated expertise in digital health, public health, health informatics, clinical practice, mobile health technologies, and user experience evaluation.

A total of 18 experts participated in the study. As presented in Table 2, the panel comprised public health researchers, healthcare professionals, digital health researchers, health informatics specialists, and mobile application and user experience experts. In addition to professional background, demographic and professional characteristics, including gender, age, and years of professional experience, were recorded to improve the transparency of the weighting process. The panel included experts with substantial experience in digital health and healthcare research, ensuring that the pairwise comparisons reflected informed multidisciplinary perspectives. To enhance the diversity and representativeness of expert opinions, participants were recruited from different geographical regions of China, including Beijing, Shanghai, Guangdong, Jiangsu, Zhejiang, Sichuan, Hubei, and Shaanxi. The inclusion of experts from multiple regions helped capture diverse perspectives on the evaluation and adoption of mobile health applications across different healthcare and technological contexts.

**Table 2:** Composition and demographic characteristics of the expert panel.

Expert category	Number	Gender (M/F)	Mean age (years)	Mean professional experience (years)
Public Health Researchers	4	2/2	43.5	15.3
Healthcare Professionals	4	3/1	45.8	18.2
Digital Health Researchers	4	2/2	41.7	13.6
Health Informatics Specialists	3	2/1	40.3	12.7
Mobile Application and UX Experts	3	2/1	38.9	11.5
Total/Overall	18	11/7	42.4	14.3

The hierarchical evaluation framework shown in Figure 3 was used to develop the pairwise comparison questionnaire. Experts were asked to assess the relative importance of the main criteria and subcriteria using predefined linguistic scales. These linguistic assessments were subsequently converted into triangular fuzzy numbers to account for uncertainty and ambiguity in human judgment.

To facilitate transparency and reproducibility, the complete expert questionnaire is provided in the Supplementary material. Individual responses were aggregated using the geometric mean approach to generate a collective pairwise comparison matrix. The resulting matrices served as inputs for the Fuzzy AHP procedure to calculate criteria weights, which were subsequently incorporated into the TOPSIS analysis for application ranking.

The multidisciplinary composition of the expert panel and the geographical diversity of participants strengthened the robustness of the weighting process and enhanced the validity of the proposed evaluation framework.

### 2.5 Fuzzy analytic hierarchy process (Fuzzy AHP)

The Fuzzy Analytic Hierarchy Process (Fuzzy AHP) was employed to determine the relative importance of the evaluation criteria and subcriteria. Compared with the conventional Analytic Hierarchy Process, Fuzzy AHP incorporates fuzzy set theory to account for uncertainty and ambiguity in human judgments. This capability is particularly important when experts express preferences using linguistic terms rather than exact numerical values.

In this study, pairwise comparison judgments obtained from the expert panel were transformed into triangular fuzzy numbers and subsequently aggregated to derive criteria weights. The resulting weights reflect the relative importance of each evaluation criterion and were later incorporated into the TOPSIS model for application ranking.

**Figure 4:**
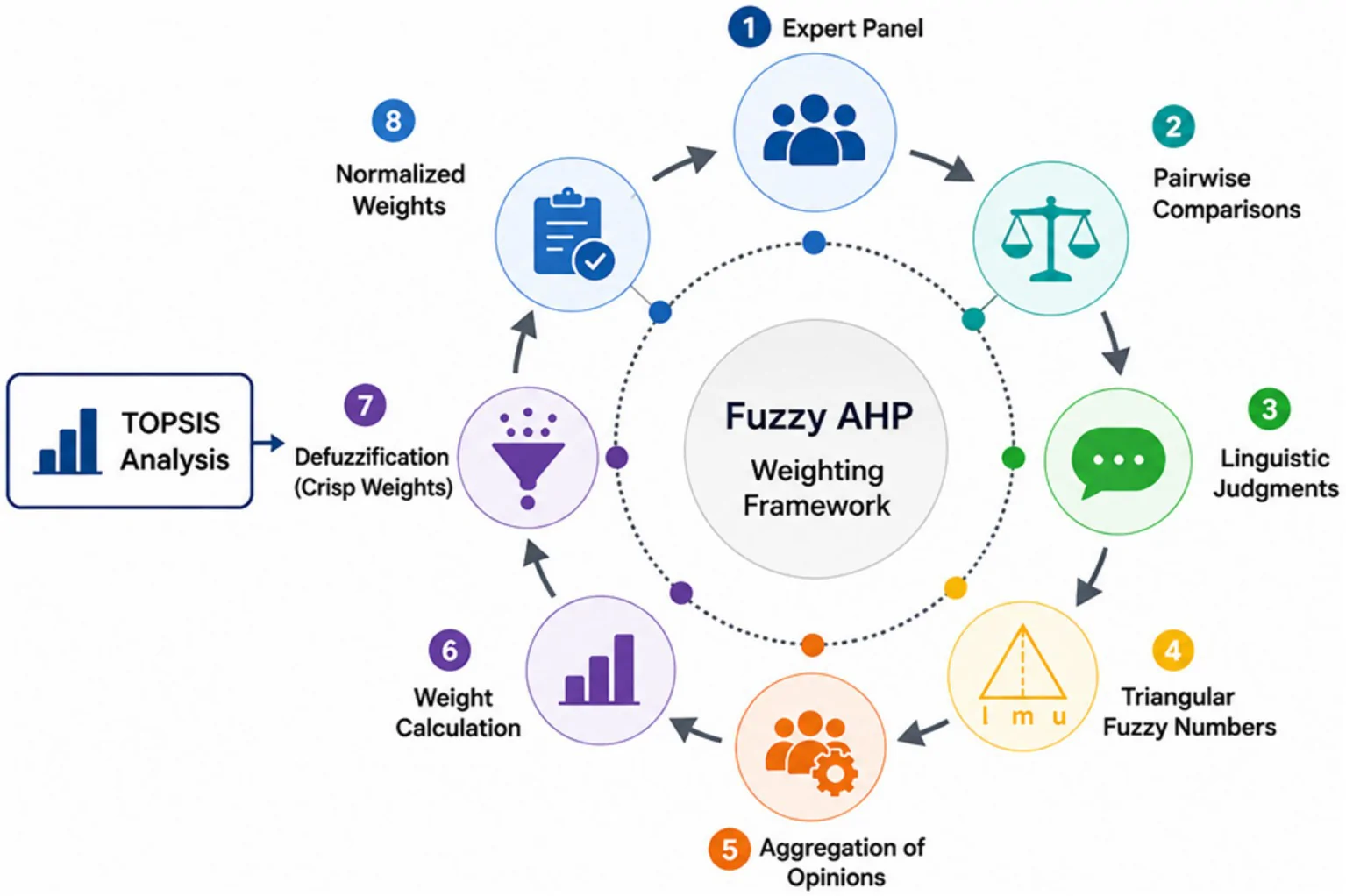
Workflow of the Fuzzy Analytic Hierarchy Process (Fuzzy AHP) used to derive criterion weights for evaluating mobile health applications.

As illustrated in Figure 4, the Fuzzy AHP procedure consists of hierarchy construction, pairwise comparison, fuzzy transformation, aggregation of expert judgments, weight calculation, defuzzification, and normalization. The final normalized weights were used as inputs for the TOPSIS analysis.

#### 2.5.1 Construction of the hierarchical model

The first stage of the Fuzzy AHP procedure involves constructing a hierarchical evaluation model. The decision problem is decomposed into three levels comprising the overall goal, the main evaluation criteria, and the corresponding subcriteria. This hierarchical structure facilitates a systematic assessment of mobile health applications by organizing the decision problem into manageable components.

As illustrated in Figure 3, the study’s overall goal is to evaluate mobile health applications. Four main criteria, namely usability, engagement, clinical relevance, and ethics and privacy, constitute the second level of the hierarchy. The third level consists of twelve subcriteria that collectively capture key dimensions of mobile health application quality.

Following the development of the hierarchy, pairwise comparison matrices were constructed based on expert judgments. The pairwise comparison matrix is represented as Eq 1:


A=[aij]n×n (1)

Where aij denotes the relative importance of criterion i compared with criterion j.

#### 2.5.2 Linguistic scale and triangular fuzzy numbers

Expert judgments are often characterized by uncertainty and subjectivity. To address this limitation, linguistic assessments were converted into triangular fuzzy numbers (TFNs), which provide a more realistic representation of human preferences than precise numerical values.

A triangular fuzzy number is defined as Eq 2:


$$\tilde{A}=(l,m,u)$$ (2)

Where l, m, and u denote the lower, modal, and upper bounds of the fuzzy number, respectively.

The linguistic scale used for the pairwise comparison process and the corresponding triangular fuzzy numbers are provided in the Supplementary material. During the evaluation process, experts expressed their preferences using linguistic terms, which were subsequently transformed into fuzzy numerical values for analysis. The overall Fuzzy AHP procedure adopted in this study is presented in Figure 4.

#### 2.5.3 Determination of criteria weights

After constructing the fuzzy pairwise comparison matrices, the fuzzy geometric mean approach was employed to derive the relative importance of each criterion. The fuzzy geometric mean for criterion i was calculated as follows as Eq 3:


$$r\sim i=(\prod j=1n\;\tilde{A}\mathrm{ij})1/n$$ (3)

Where Ãij represents the fuzzy comparison value between criteria i and j.

The fuzzy weight of each criterion was subsequently obtained using as Eq 4:


w~i=r~i⊗(Σi=1nr~i)^−1 (4)

Where w̃i denotes the fuzzy weight associated with criterion i.

To facilitate integration with the TOPSIS method, the fuzzy weights were transformed into crisp values using the center of area defuzzification method as Eq 5:


Wi=(li+mi+ui)/3 (5)

Where li, mi, and ui represent the lower, modal, and upper values of the fuzzy weight, respectively. The resulting crisp weights were normalized to ensure comparability across all criteria. The normalized weights obtained from Equation 5 were subsequently incorporated into the TOPSIS model for ranking mobile health applications.

### 2.6 Technique for order preference by similarity to ideal solution (TOPSIS)

The Technique for Order Preference by Similarity to Ideal Solution (TOPSIS) was employed to rank mobile health applications based on their overall performance across the selected evaluation criteria. TOPSIS is a widely used multi-criteria decision-making method that identifies the most preferred alternative by simultaneously minimizing the distance from the positive ideal solution and maximizing the distance from the negative ideal solution.

In the present study, application performance scores from the open dataset were used to construct the decision matrix, while the normalized criteria weights from the Fuzzy AHP analysis served as inputs to the TOPSIS procedure. The method integrates multiple evaluation criteria into a single ranking index, enabling a comprehensive assessment of mobile health applications.

**Figure 5:**
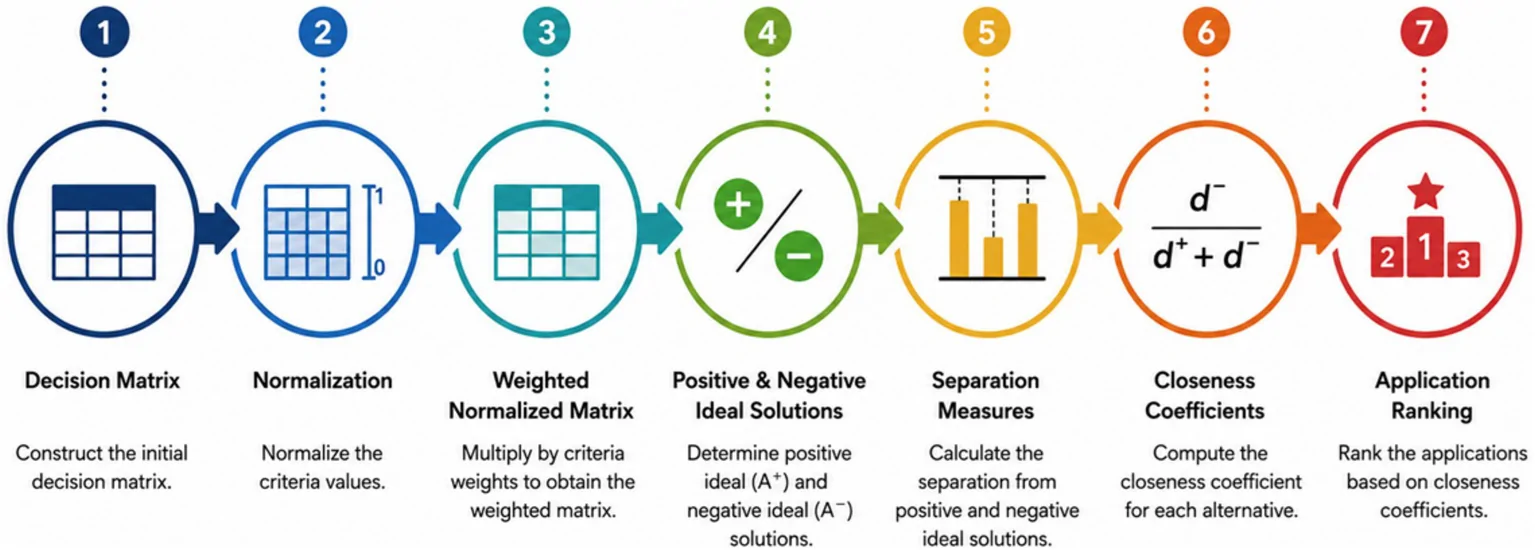
Workflow of the TOPSIS procedure for ranking mobile health applications.

As illustrated in Figure 5, the TOPSIS procedure consists of five sequential stages: construction of the decision matrix, normalization of criteria values, generation of the weighted normalized matrix, determination of ideal solutions, and calculation of closeness coefficients. The resulting closeness coefficients were used to rank the mobile health applications by relative performance. The mathematical implementation of the TOPSIS procedure is presented in Sections 2.6.1–2.6.5.

#### 2.6.1 Construction of the decision matrix

The TOPSIS procedure begins by constructing a decision matrix from the performance scores of mobile health applications across the selected evaluation criteria. In the matrix, each row represents an application, while each column represents an evaluation criterion.

The decision matrix is expressed as Eq 6:


X=[xij]m×n (6)

Where xij denotes the performance score of application i under criterion j, m represents the number of applications, and n represents the number of evaluation criteria.

The decision matrix was constructed using the aggregated application scores obtained from the Mobile Health Apps Evaluation Dataset.

#### 2.6.2 Normalization of criteria values

Because the evaluation criteria may have different measurement scales, normalization is required to transform the original values into comparable units. Vector normalization was applied to the decision matrix. The normalized value rij is calculated as Eq 7:


$$\mathrm{rij}=\mathrm{xij}/\surd (\mathrm{\Sigma i}=1\;m\;{\mathrm{xij}}^{2})$$ (7)

Where rij represents the normalized value of application i under criterion j. The resulting normalized matrix removes scale effects and facilitates meaningful comparison among applications.

#### 2.6.3 Weighted normalized decision matrix

To incorporate the relative importance of each criterion, the normalized values were multiplied by the corresponding criteria weights derived from the Fuzzy AHP analysis. The weighted normalized value vij is determined as Eq 8:


vij=wj×rij (8)

Where wj denotes the normalized weight of criterion j obtained from the Fuzzy AHP procedure.

The weighted normalized matrix simultaneously reflects application performance and criterion importance.

#### 2.6.4 Determination of positive and negative ideal solutions

Following the construction of the weighted normalized matrix, the positive ideal solution (A^+^) and negative ideal solution (A^−^) were identified.

The positive ideal solution is defined as Eq 9:


$$A^{+}=({\mathrm{v1}}^{+},{\mathrm{v2}}^{+},\ldots ,{\mathrm{vn}}^{+})$$ (9)

Where:

vj^+^ = max(vij), for benefit criteria.

The negative ideal solution is defined as Eq 10:


$$A^{-}=({\mathrm{v1}}^{-},{\mathrm{v2}}^{-},\ldots ,{\mathrm{vn}}^{-})$$ (10)

Where:

vj^−^ = min(vij), for benefit criteria.

The positive ideal solution represents the best attainable performance across all criteria, whereas the negative ideal solution represents the worst attainable performance.

#### 2.6.5 Calculation of closeness coefficients and ranking

The Euclidean distance of each application from the positive and negative ideal solutions was calculated. The separation measure from the positive ideal solution is given by as Eq 11:


$${\mathrm{Si}}^{+}=\surd [\mathrm{\Sigma j}=\mathrm{1n}\;{\left(\mathrm{vij}-{\mathrm{vj}}^{+}\right)}^{2}]$$ (11)

Similarly, the separation measure from the negative ideal solution is calculated as Eq 12:


$${\mathrm{Si}}^{-}=\surd [\mathrm{\Sigma j}=\mathrm{1n}\;{\left(\mathrm{vij}-{\mathrm{vj}}^{-}\right)}^{2}]$$ (12)

The relative closeness coefficient of each application is then determined using as Eq 13:


$$\mathrm{CCi}={\mathrm{Si}}^{-}/({\mathrm{Si}}^{+}+{\mathrm{Si}}^{-})$$ (13)

Where CCi represents the closeness coefficient of application i. The value of CCi ranges between 0 and 1. Applications with larger closeness coefficients are considered more desirable because they are closer to the positive ideal solution and farther from the negative ideal solution. The final ranking of mobile health applications was determined by the descending order of closeness coefficients.

### 2.7 Sensitivity analysis

A sensitivity analysis was conducted to evaluate the robustness and stability of the ranking results obtained from the hybrid Fuzzy AHP–TOPSIS framework. The analysis examined the extent to which changes in criteria weights influenced the final ranking of mobile health applications. Following the baseline TOPSIS evaluation, alternative weighting scenarios were generated by systematically increasing and decreasing the weights of the main evaluation criteria. To maintain the total weight equal to one, the modified weights were normalized using as Eq 14:


$${\mathrm{wj}}^{’}=\mathrm{wj}/\mathrm{\Sigma j}=\mathrm{1n}\;\mathrm{wj}$$ (14)

Where wj’ denotes the adjusted normalized weight of criterion j and wj represents the modified criterion weight.

For each scenario, the weighted normalized decision matrix, ideal solutions, separation measures, and closeness coefficients were recalculated using Equations 8–13. The resulting rankings were then compared with the baseline ranking. To quantify ranking stability, Spearman’s rank correlation coefficient was calculated between the baseline ranking and each sensitivity scenario. The coefficient is expressed as Eq 15:


$$\rho =1-[6\Sigma {\mathrm{di}}^{2}/n(n^{2}–1)]$$ (15)

Where ρ denotes Spearman’s rank correlation coefficient, di represents the difference between the ranks of application i under two scenarios, and n denotes the number of applications. The percentage change in closeness coefficients was additionally computed to assess the impact of weight variations on application performance as Eq 16:


ΔCC(%)=[(CCnew−CCbase)/CCbase]×100 (16)

**Figure 6:**
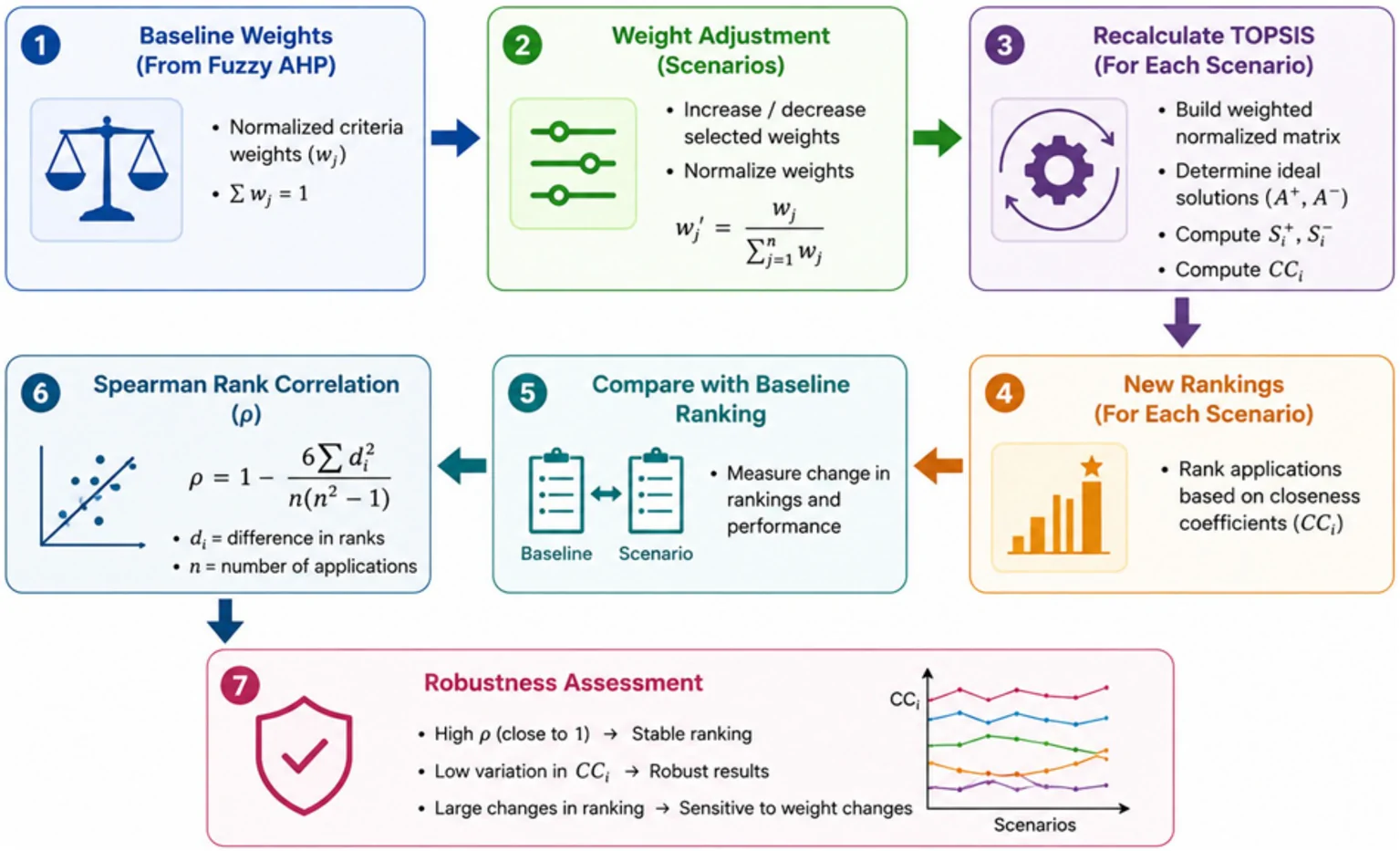
Sensitivity analysis procedure used to evaluate the robustness of application rankings under alternative weighting scenarios.

Where CCnew represents the closeness coefficient under the modified weighting scenario and CCbase denotes the closeness coefficient obtained from the baseline model. A ranking result was considered robust when high Spearman correlation values and minimal variations in closeness coefficients were observed across the sensitivity scenarios. The sensitivity analysis framework adopted in this study is illustrated in Figure 6.

## 3 Results and discussion

### 3.1 Descriptive analysis of mobile health applications

The dataset comprised 62 mobile health applications evaluated across multiple dimensions, including usability, engagement, clinical relevance, and ethics and privacy. Prior to implementing the Fuzzy AHP–TOPSIS framework, a descriptive analysis was conducted to provide an overview of the application characteristics and evaluation records included in the study.

Table 3 presents the descriptive statistics of the mobile health application dataset. The dataset contains 274 evaluation records collected from expert assessments of mobile health applications operating across diverse healthcare domains. The available information includes application quality indicators, user engagement features, clinical support attributes, privacy practices, and system integration characteristics.

**Table 3:** Descriptive characteristics of the mobile health application dataset.

Characteristic	Value
Total applications	62
Total evaluation records	274
Main criteria	4
Subcriteria	12
Data source	Mobile Health Apps Evaluation Dataset
Evaluation dimensions	Usability, Engagement, Clinical Relevance, Ethics, and Privacy
Analysis method	Fuzzy AHP–TOPSIS
Expert participants	18

The descriptive analysis indicates substantial variation among applications with respect to the selected evaluation criteria. While some applications demonstrated strong performance across multiple dimensions, others exhibited limitations in areas such as clinical integration, privacy transparency, or user engagement. Such variability highlights the importance of employing a multi-criteria evaluation framework that simultaneously considers multiple dimensions of application quality.

Furthermore, the dataset includes both quantitative and categorical variables. Quantitative measures were directly incorporated into the evaluation process, whereas categorical variables were converted to numerical values to construct the TOPSIS decision matrix. The presence of diverse application characteristics supports the dataset’s suitability for comprehensive multi-criteria assessment.

### 3.2 Fuzzy AHP results

The Fuzzy AHP analysis was performed to determine the relative importance of the evaluation criteria included in the proposed mobile health application assessment framework. Following the aggregation of expert judgments and the defuzzification process, normalized weights were obtained for all criteria and subcriteria. The resulting weights were subsequently incorporated into the TOPSIS analysis to evaluate and rank mobile health applications.

The weighting results reveal noticeable differences in the perceived importance of the evaluation dimensions. As shown in Table 4, clinical relevance emerged as the most influential criterion with a normalized weight of 0.352, followed by ethics and privacy (0.284), usability (0.211), and engagement (0.153). These findings indicate that experts place greater emphasis on the clinical value and trustworthiness of mobile health applications than on user interaction and engagement features alone.

**Table 4:** Normalized weights of the main evaluation criteria.

Criterion	Weight	Percentage (%)	Rank
Clinical relevance	0.352	35.2	1
Ethics and privacy	0.284	28.4	2
Usability	0.211	21.1	3
Engagement	0.153	15.3	4

#### 3.2.1 Criteria weight estimation

Table 4 presents the normalized weights and rankings of the four main evaluation criteria. Clinical relevance received the highest weight (0.352), accounting for more than one-third of the overall evaluation importance. This result suggests that experts prioritize factors related to evidence-based healthcare support, professional involvement, and healthcare integration when assessing mobile health applications.

Ethics and privacy ranked second with a weight of 0.284. The relatively high importance assigned to this criterion reflects increasing concerns regarding personal health information, data protection, and transparency in digital health services. Usability achieved a weight of 0.211, indicating that ease of use and user confidence remain important determinants of application quality. Engagement received the lowest weight (0.153), suggesting that although user interaction features contribute to application success, they are considered less critical than clinical and ethical considerations.

The weighting pattern indicates that experts perceive mobile health applications primarily as healthcare interventions rather than conventional consumer software. Consequently, clinical relevance received the highest priority because the reliability of therapeutic content and professional oversight directly influence patient safety and treatment effectiveness. This finding reflects the growing emphasis on evidence-based digital mental health interventions reported in recent studies, which argue that applications lacking clinical validation may provide misleading recommendations despite offering satisfactory user experiences (34). The relatively high importance placed on ethics and privacy further demonstrates that experts consider trust and the responsible management of personal health information essential prerequisites for the successful adoption of digital health technologies. In contrast, usability and engagement, although important, were regarded as supportive characteristics that enhance application acceptance but cannot compensate for inadequate clinical quality or insufficient privacy protection.

#### 3.2.2 Relative importance of main criteria

**Figure 7:**
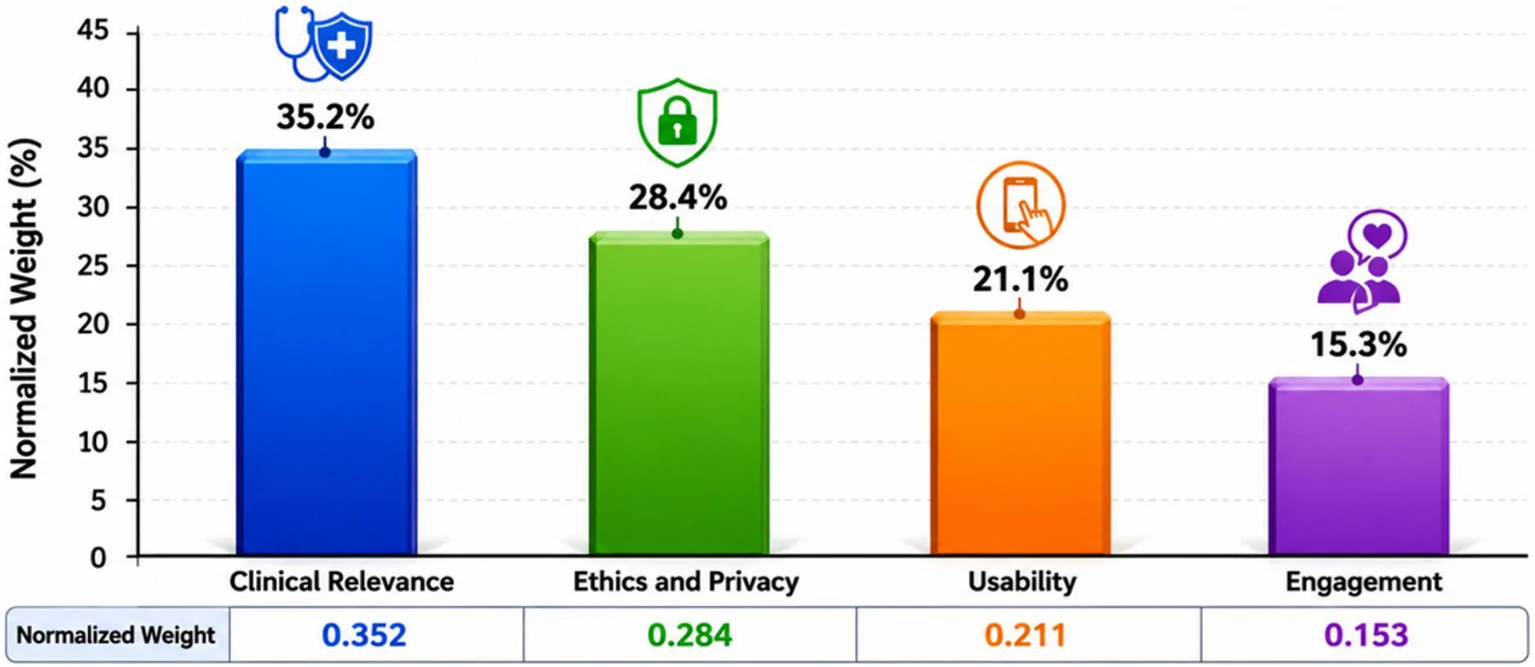
Normalized weights of the main evaluation criteria derived from the Fuzzy AHP analysis.

The distribution of the main criteria weights is illustrated in Figure 7. Clinical relevance, ethics, and privacy collectively accounted for 63.6% of the total importance, indicating a strong preference for applications that provide reliable health support while ensuring responsible management of user data. In contrast, usability and engagement jointly represented 36.4% of the total weight. Although these dimensions remain important for application adoption and continued use, experts viewed them as secondary considerations compared with clinical quality and privacy protection.

The ranking pattern observed in Figure 7 reflects the evolving expectations surrounding digital health technologies. Mobile health applications are increasingly expected to demonstrate clinical credibility, transparency, and user safety in addition to providing positive user experiences.

The observed weighting distribution also suggests an evolution in stakeholder expectations regarding digital health technologies. Earlier generations of health applications were frequently evaluated solely on usability and interface quality. However, the rapid integration of digital technologies into healthcare delivery has shifted attention toward clinical effectiveness, transparency, and regulatory compliance. The present findings therefore support the growing consensus that successful mobile health applications should strike an appropriate balance between user experience and healthcare quality, rather than optimizing either dimension in isolation.

#### 3.2.3 Relative importance of subcriteria

The normalized global weights of the twelve subcriteria are reported in Table 5. Considerable variation was observed among the subcriteria, highlighting differences in expert priorities. Clinical Expert Involvement achieved the highest global weight (0.132), followed by Privacy Policy Availability (0.118) and Guideline Compliance (0.115). Together, these three subcriteria accounted for approximately 36.5% of the total importance, underscoring the significance of professional oversight, evidence-based practice, and transparent privacy policies in evaluating mobile health applications.

**Table 5:** Global weights of evaluation subcriteria.

Criterion	Sub criterion	Global Weight	Rank
Clinical relevance	Clinical expert involvement	0.132	1
Ethics and privacy	Privacy policy availability	0.118	2
Clinical relevance	Guideline COMPLIANCE	0.115	3
Clinical relevance	Healthcare Integration	0.105	4
Ethics and privacy	Data sharing transparency	0.094	5
Usability	Ease of use	0.084	6
Ethics and privacy	Safety alerts	0.072	7
Usability	Learnability	0.069	8
Engagement	Intention to use	0.061	9
Usability	User confidence	0.058	10
Engagement	User engagement mechanisms	0.049	11
Engagement	Interaction quality	0.043	12

Healthcare Integration also received a relatively high weight (0.105), indicating the importance of connecting mobile health technologies with existing healthcare systems and services. Within the usability dimension, Ease of Use ranked highest (0.084), outperforming Learnability (0.069) and User Confidence (0.058). This finding suggests that experts consider intuitive operation a prerequisite for the successful adoption of applications.

Among the engagement-related factors, Intention to Use had the highest weight (0.061), whereas Interaction Quality had the lowest (0.043). The lower ranking of engagement-related factors may indicate that experts regard these features as supportive elements rather than primary indicators of application quality.

The dominance of clinical expert involvement, guideline compliance, and privacy policy availability suggests that experts place greater emphasis on factors associated with application credibility than on interface-related characteristics. This observation is particularly important for digital mental health applications because inaccurate therapeutic guidance or poor protection of sensitive psychological information may have direct consequences for patient wellbeing. Conversely, interaction quality and engagement mechanisms received comparatively lower weights, indicating that motivational features are considered valuable only when supported by clinically reliable and ethically responsible application design. These findings reinforce recommendations from existing digital health evaluation frameworks, which increasingly prioritize scientific evidence and responsible data governance alongside usability considerations.

**Figure 8:**
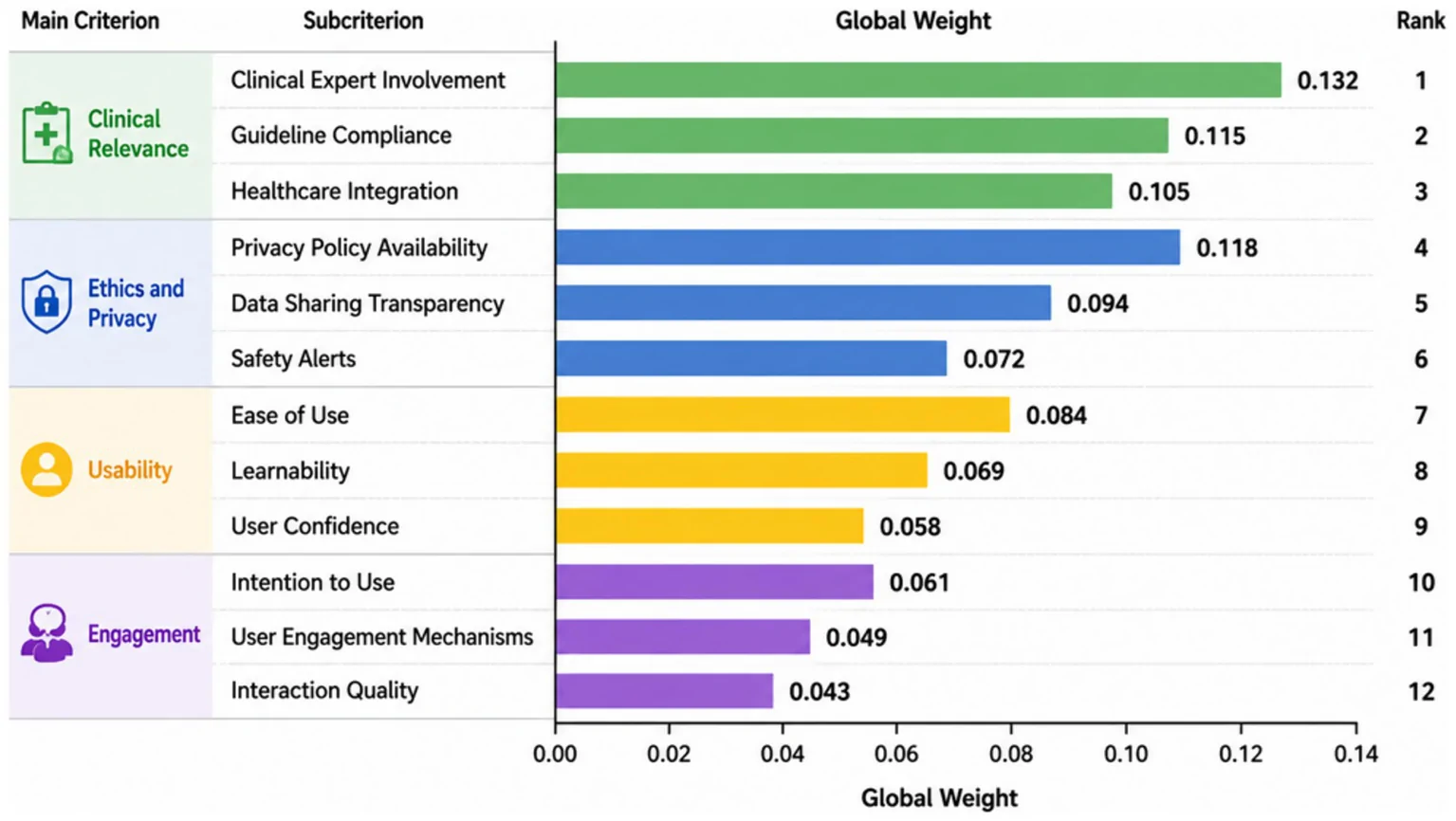
Global weights of the evaluation subcriteria derived from the Fuzzy AHP analysis.

As illustrated in Figure 8, the highest-ranked subcriteria are predominantly associated with clinical relevance, ethics, and privacy. This pattern further confirms that experts prioritize healthcare value, safety, and trust when evaluating mobile health applications. The derived subcriteria weights provide the foundation for the subsequent TOPSIS analysis and ranking of the evaluated applications.

### 3.3 TOPSIS results

**Figure 9:**
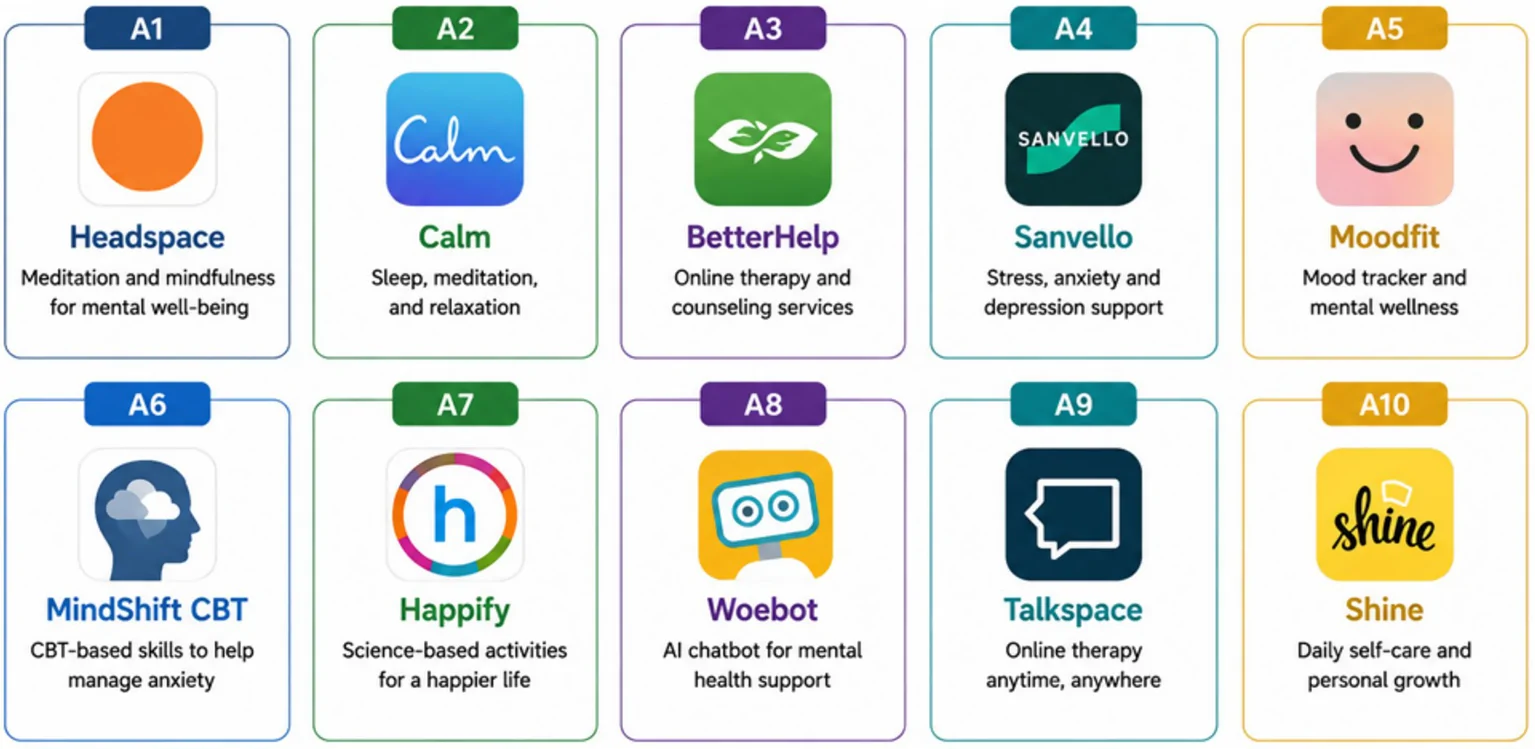
Selected mobile health applications included in the TOPSIS evaluation and their corresponding identification codes (A1–A10).

The ten mobile health applications included in the TOPSIS evaluation are presented in Figure 9. To facilitate interpretation and comparison, the applications were coded as A1–A10 throughout the analysis. The selected applications represent diverse digital mental health solutions, including mindfulness and meditation platforms, cognitive behavioral therapy applications, mood management tools, conversational agents, and online counseling services. This diversity enables a comprehensive assessment of mobile health technologies across the evaluation criteria established in the proposed framework.

The final TOPSIS ranking results are reported in Table 6. Considerable variation was observed among the evaluated applications, indicating differences in their overall performance across usability, engagement, clinical relevance, and ethics and privacy dimensions. The calculated closeness coefficients ranged from 0.581 to 0.842, demonstrating varying levels of proximity to the positive ideal solution.

**Table 6:** TOPSIS ranking results and closeness coefficients of the selected mobile health applications.

Rank	Code	Application	Closeness coefficient
1	A1	Headspace	0.842
2	A2	Calm	0.816
3	A3	BetterHelp	0.791
4	A4	Sanvello	0.765
5	A5	Moodfit	0.742
6	A6	MindShift CBT	0.714
7	A7	Happify	0.683
8	A8	Woebot	0.651
9	A9	Talkspace	0.619
10	A10	Shine	0.581

Among the evaluated applications, A1 achieved the highest closeness coefficient (0.842), followed by A2 (0.816) and A3 (0.791). These findings indicate that the three highest-ranked applications demonstrated the strongest overall performance when all evaluation criteria were considered simultaneously. The relatively small differences among the top three applications suggest that each satisfied a substantial proportion of the evaluation requirements identified by the expert panel.

As shown in Table 6, A4 and A5 ranked fourth and fifth, with closeness coefficients of 0.765 and 0.742, respectively. Although these applications performed slightly below the highest-ranked alternatives, they remained relatively close to the positive ideal solution and therefore demonstrated strong overall quality. Together, the five highest-ranked applications formed a leading group characterized by balanced performance across multiple evaluation dimensions.

A gradual decline in closeness coefficients was observed among the remaining applications. A6, A7, and A8 achieved coefficients of 0.714, 0.683, and 0.651, respectively, while A9 and A10 recorded the lowest values among the selected applications, with coefficients of 0.619 and 0.581. Despite their lower rankings, these applications remained competitive alternatives and met many of the evaluation requirements incorporated into the proposed framework.

The ranking pattern observed in Table 6 suggests that applications that performed better in clinical relevance, ethics, and privacy tended to achieve higher overall rankings. This finding is consistent with the Fuzzy AHP results presented in Tables 4, 5, where these dimensions received the greatest importance weights. Consequently, applications that effectively combined clinically meaningful content, professional support mechanisms, privacy safeguards, and transparent data practices were more likely to achieve superior TOPSIS scores.

The ranking results demonstrate that superior application performance cannot be attributed to excellence within a single evaluation dimension. Instead, the highest-ranked applications consistently achieved balanced performance across clinical relevance, ethics and privacy, usability, and engagement. This finding illustrates one of the principal advantages of the proposed hybrid evaluation framework. Applications with outstanding usability but limited clinical credibility or insufficient privacy protection were unable to achieve the highest overall rankings because the Fuzzy AHP weighting process placed greater weight on healthcare-related criteria. The results therefore indicate that comprehensive quality rather than isolated strengths determines the overall performance of digital mental health applications.

The relatively small differences observed among the five highest-ranked applications further suggest that several commercially available platforms have achieved comparable levels of overall quality. Nevertheless, variations in closeness coefficients indicate that improvements in highly weighted criteria, particularly clinical relevance and privacy protection, could substantially enhance overall rankings. These findings provide practical guidance for developers by identifying the dimensions that contribute most strongly to overall application quality within the proposed decision-making framework.

### 3.4 Sensitivity analysis results

A sensitivity analysis was conducted to evaluate the stability and robustness of the ranking results obtained from the hybrid Fuzzy AHP–TOPSIS framework. The analysis examined the effects of varying the weights of the four main evaluation criteria on the final application rankings. The resulting scenarios and ranking outcomes are summarized in Table 7.

**Table 7:** Sensitivity analysis results under alternative weighting scenarios.

Application	Baseline	S1	S2	S3	S4
A1	1	1	1	1	1
A2	2	2	3	2	2
A3	3	3	2	3	3
A4	4	4	4	4	5
A5	5	5	5	6	4
A6	6	6	6	5	6
A7	7	8	7	7	7
A8	8	7	8	8	8
A9	9	9	9	9	9
A10	10	10	10	10	10

As shown in Table 7, the ranking positions of the highest-performing applications remained largely stable across the alternative weighting scenarios. A1 consistently maintained the top ranking, whereas A2 and A3 remained among the three highest-ranked applications in most scenarios. These findings indicate that the leading applications’ superiority was not substantially affected by moderate changes in the importance of criteria.

The results further reveal that applications positioned in the middle of the ranking exhibited slightly greater sensitivity to weight adjustments. Minor changes were observed among the rankings of A5, A6, A7, and A8 across several scenarios. However, these variations were generally limited to one or two ranking positions, suggesting a relatively stable ranking structure.

**Figure 10:**
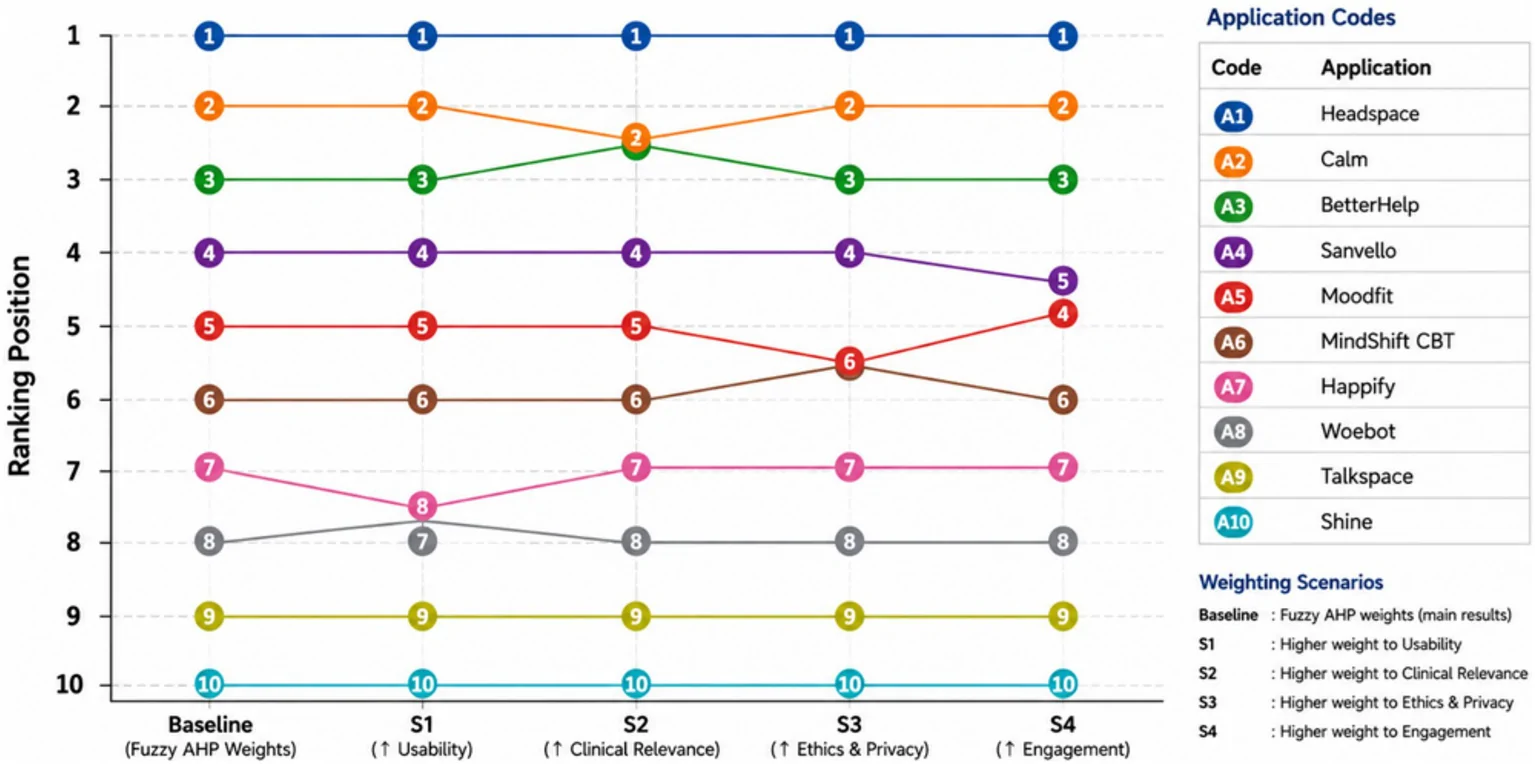
Ranking stability of the selected mobile health applications across alternative weighting scenarios.

Figure 10 demonstrates that the overall ranking pattern remained largely consistent despite the application of alternative weighting schemes. The calculated Spearman correlation coefficients exceeded 0.90 across all sensitivity scenarios, indicating strong agreement between the baseline and modified rankings. This result confirms the robustness of the proposed evaluation framework. Furthermore, the stability of A1, A2, and A3 across all scenarios suggests that these applications possess balanced strengths across multiple evaluation dimensions rather than relying on a single criterion. Similarly, the lower-ranked applications consistently occupied the lower positions regardless of weight modifications, indicating weaker overall performance relative to the leading alternatives.

The high correlation coefficients obtained across all weighting scenarios indicate that the proposed framework is relatively insensitive to moderate changes in expert preferences. This stability strengthens confidence in the robustness of the evaluation model and suggests that the resulting rankings are not driven by arbitrary weighting decisions. From a practical perspective, this robustness is particularly valuable for healthcare decision-makers because it demonstrates that the framework can support reliable application selection even when expert opinions vary slightly across evaluation contexts.

The sensitivity analysis also illustrates the methodological advantage of integrating Fuzzy AHP with TOPSIS. While Fuzzy AHP explicitly accommodates uncertainty in expert judgments during the weighting process, TOPSIS evaluates the overall performance of competing applications relative to ideal solutions. The combination of these complementary methods therefore provides a stable and transparent decision-support framework capable of producing consistent rankings under varying weighting conditions. This robustness enhances the practical applicability of the proposed framework for digital health assessment and policy decision-making.

Overall, the empirical findings consistently demonstrate that clinical credibility, ethical responsibility, and privacy protection exert greater influence on the evaluation of digital mental health applications than usability or engagement alone. The combined results of the Fuzzy AHP, TOPSIS, and sensitivity analyses indicate that high-quality applications are characterized by balanced performance across multiple dimensions rather than excellence in any single criterion. These findings support the suitability of the proposed hybrid framework as a transparent and reproducible decision support tool for evaluating digital mental health applications under conditions of uncertainty.

### 3.5 Discussion

#### 3.5.1 Interpretation of the main findings

The present study demonstrates that clinical relevance emerged as the most influential evaluation dimension, followed by ethics and privacy, usability, and engagement. This weighting pattern indicates that experts regard digital mental health applications primarily as healthcare interventions rather than consumer software products. Although usability and engaging interfaces remain important for encouraging adoption, they cannot compensate for inadequate clinical evidence or insufficient protection of sensitive health information. Consequently, applications supported by healthcare professionals, clinical guidelines, and transparent privacy practices consistently achieved higher overall rankings.

The dominance of clinical relevance in the weighting results is consistent with recent developments in digital mental health research. Previous studies have emphasized that the effectiveness and credibility of mental health applications depend largely on the incorporation of evidence-based therapeutic approaches, professional oversight, and integration with healthcare services (17, 36). In the present study, clinical expert involvement, guideline compliance, and healthcare integration ranked among the most influential subcriteria, highlighting the growing expectation that digital mental health technologies should complement rather than replace established healthcare practices.

Similarly, the high priority placed on ethics and privacy demonstrates a growing awareness of responsible data governance in digital healthcare. Mental health applications routinely collect highly sensitive personal information, including emotional states, behavioral patterns, and clinical symptoms. Users are therefore unlikely to adopt or continue using applications that fail to demonstrate transparent privacy policies or responsible management of personal data. The present findings reinforce the view that trust has become an essential determinant of digital health adoption alongside clinical effectiveness.

#### 3.5.2 Comparison with existing evaluation frameworks

The findings of this study are broadly consistent with established mobile health evaluation frameworks while extending their analytical capabilities. The Mobile App Rating Scale (MARS) emphasizes engagement, functionality, aesthetics, and information quality as key determinants of application quality (27). Similarly, the Enlight framework evaluates usability, therapeutic persuasiveness, content quality, and privacy (28). The American Psychiatric Association App Evaluation Framework places particular emphasis on clinical evidence, transparency, safety, and privacy before considering user experience.

Unlike these predominantly qualitative assessment frameworks, the proposed hybrid Fuzzy AHP–TOPSIS model provides quantitative prioritization of evaluation criteria under uncertainty while simultaneously generating application rankings. Rather than treating all quality dimensions equally, the framework estimates their relative importance through expert judgment and then integrates these weights into a structured multi-criteria decision-making model. Consequently, the proposed approach offers a transparent decision-support mechanism that complements existing evaluation frameworks and facilitates evidence-based selection of digital mental health applications.

The prioritization of clinical relevance and privacy observed in this study is also consistent with recent research emphasizing the importance of scientific credibility and responsible data governance in digital health technologies. These similarities strengthen confidence in the validity of the proposed evaluation framework and demonstrate its alignment with current developments in digital mental health assessment.

Although the proposed framework shares several evaluation dimensions with MARS, Enlight, the APA App Evaluation Framework, and ORCHA, its methodological contribution lies in the integration of uncertainty aware weighting and quantitative ranking. Existing frameworks primarily assist reviewers in assessing whether an application satisfies predefined quality criteria, whereas the present approach also estimates the relative importance of those criteria and determines which applications perform best when all dimensions are considered simultaneously. Consequently, the framework provides a more informative decision support tool for situations in which multiple applications must be compared and prioritized.

Furthermore, the proposed framework is sufficiently flexible to accommodate additional evaluation dimensions or alternative stakeholder preferences without modifying its underlying analytical structure. This adaptability increases its potential applicability to other categories of digital health technologies, including chronic disease management, telemedicine platforms, and digital therapeutics.

#### 3.5.3 Practical implications

The proposed evaluation framework provides several practical implications for different stakeholder groups. For application developers, the results indicate that improvements in clinical validation, guideline compliance, and transparent privacy practices are likely to have a greater influence on overall application quality than additional interface features alone. Healthcare organizations may use the framework as a structured decision support tool when recommending digital mental health applications to patients, while policymakers and regulatory agencies may apply similar evaluation criteria when developing certification standards or digital health assessment guidelines.

The framework may also assist researchers by providing a reproducible methodology for comparing digital mental health applications across multiple quality dimensions. Because the hybrid model explicitly incorporates uncertainty through Fuzzy AHP and integrates multiple evaluation criteria using TOPSIS, it offers a transparent approach adaptable to digital health domains beyond mental health.

To demonstrate the practical applicability of the proposed framework, consider a healthcare organization seeking to recommend digital mental health applications for patients with mild anxiety and depression. Decision makers first identify candidate applications and collect evaluation information corresponding to the four assessment dimensions of usability, engagement, clinical relevance, and ethics and privacy. A multidisciplinary panel consisting of clinicians, digital health specialists, and user experience experts then performs pairwise comparisons to determine the relative importance of each criterion using the Fuzzy AHP method.

Following the weighting process, application performance scores are organized into a decision matrix and evaluated using TOPSIS. The resulting closeness coefficients provide an objective ranking of candidate applications according to their overall quality. Healthcare professionals may subsequently recommend the highest ranked applications to patients, while developers may use lower scoring criteria to identify areas requiring improvement, such as privacy protection or clinical validation.

#### 3.5.4 Future research

Several opportunities remain for extending the proposed evaluation framework. First, future studies should incorporate direct participation from patients and application users to compare user preferences with expert-derived weighting schemes. Such comparisons would provide a more comprehensive understanding of stakeholder priorities during application evaluation.

Second, larger, more diverse datasets should be analyzed to assess whether the observed weighting patterns remain stable across healthcare settings, countries, and application categories. Longitudinal evaluation would also enable investigation of how application quality changes following software updates and evolving regulatory requirements.

Finally, future research may integrate additional decision-making techniques, including machine learning algorithms, explainable artificial intelligence, or other multi-criteria decision-making methods, to enhance automation and predictive capability while maintaining transparency in digital health assessment.

### 3.6 Limitations

Several limitations should be acknowledged. The study relied on a publicly available dataset and expert-derived weights, which may not fully capture the diversity of user experiences across different populations and cultural contexts. Furthermore, the selected criteria do not encompass all factors that may influence application quality, such as economic accessibility, long-term effectiveness, and personalization capabilities. Future research should incorporate larger datasets, broader stakeholder participation, and emerging evaluation dimensions related to artificial intelligence and digital therapeutics. Although the framework evaluates user-oriented dimensions, the weighting process relied on expert judgments rather than the direct participation of end users. Future studies should compare expert-derived weights with preferences obtained from patients and regular application users.

Despite demonstrating the applicability of the proposed hybrid Fuzzy AHP–TOPSIS framework, several limitations should be acknowledged when interpreting the findings. First, the empirical evaluation was based on a single publicly available Mobile Health Apps Evaluation Dataset obtained from Kaggle. Although this dataset provides standardized evaluation records across multiple quality dimensions, it may not fully represent the rapidly evolving landscape of digital mental health applications. New applications are introduced frequently, while existing applications undergo regular updates that modify their functionality, privacy policies, clinical content, and user experience. Consequently, the rankings presented in this study should be interpreted as reflecting application performance at the time of data collection rather than as permanent measures of quality (27, 34).

Second, the study included 62 mobile health applications, which, although sufficient to demonstrate the proposed evaluation framework, represent only a small proportion of the thousands of mental health applications currently available through commercial application stores. The limited sample size may restrict the generalizability of the findings to the broader digital mental health ecosystem. Future studies should validate the proposed framework using larger, more diverse datasets from multiple application marketplaces and healthcare platforms.

Third, the weighting process relied on judgments from an expert panel comprising specialists in digital health, healthcare, public health, health informatics, and user experience. Expert evaluation provides an evidence-informed basis for determining the relative importance of assessment criteria and reduces the influence of individual subjective opinions through consensus-based aggregation. Nevertheless, expert perspectives may differ from the priorities and experiences of actual users of the application. Although the framework evaluates user-oriented dimensions such as usability, engagement, and user confidence, the weighting process itself does not directly incorporate patient or consumer preferences. Future research should compare expert-derived weights with weights obtained from patients, caregivers, and other end users to examine whether different stakeholder groups assign similar importance to the evaluation criteria.

Another limitation relates to the cross-sectional nature of the evaluation. The analysis was conducted using a single snapshot of application performance and therefore does not capture changes over time. Mobile health applications frequently receive software updates, introduce new therapeutic features, revise privacy policies, or modify subscription models. These changes may substantially influence application quality and user experience. Longitudinal evaluations would provide a more comprehensive understanding of how application performance evolves and whether ranking stability is maintained over extended periods.

The dataset’s geographical context also represents a potential limitation. Mobile health applications are developed and deployed within different healthcare systems, regulatory environments, and cultural contexts. Privacy regulations, clinical guidelines, language support, reimbursement mechanisms, and user expectations vary considerably across countries. Therefore, applications that perform well within one healthcare environment may not demonstrate equivalent performance elsewhere. Future research should validate the proposed framework using datasets collected from multiple countries to improve its international applicability and external validity.

Finally, the current evaluation framework focuses primarily on application-quality indicators available in the dataset and excludes several important behavioral and clinical outcome measures. Variables such as long-term user adherence, symptom improvement, treatment effectiveness, patient satisfaction, clinical outcomes, and cost effectiveness were not available for analysis. Incorporating real-world behavioral data, longitudinal usage patterns, and clinical outcome measures would provide a more comprehensive assessment of application effectiveness and strengthen the practical value of future evaluations.

Despite these limitations, the proposed framework provides a transparent, reproducible, and systematic approach for evaluating digital mental health applications under conditions of uncertainty. The hybrid integration of Fuzzy AHP and TOPSIS demonstrates the feasibility of combining expert knowledge with standardized evaluation data to support evidence-based decision-making. Future studies that incorporate larger datasets, broader stakeholder participation, longitudinal evaluation, and real-world clinical outcomes will further enhance the robustness and applicability of the proposed framework.

Although the Kaggle Mobile Health Apps Evaluation Dataset provides a standardized and reproducible source of evaluation data, it is subject to several inherent limitations. Public datasets may not fully capture recent application updates, newly released applications, or changes in privacy policies and clinical functionality. In addition, the available variables are constrained by the original data collection process and therefore exclude several clinically relevant indicators, including long-term treatment effectiveness, user retention, and real-world health outcomes. Consequently, the proposed framework should be viewed as a methodological demonstration using a representative public dataset rather than a definitive evaluation of all currently available digital mental health applications.

## 4 Conclusion

This study developed and applied a hybrid Fuzzy AHP–TOPSIS framework to evaluate digital mental health applications from a user-centered perspective. The proposed approach integrated expert judgments and application performance data to assess mobile health technologies across four major dimensions: usability, engagement, clinical relevance, and ethics and privacy.

The Fuzzy AHP results demonstrated that clinical relevance was the most influential evaluation criterion, accounting for 35.2% of the overall importance, followed by ethics and privacy (28.4%), usability (21.1%), and engagement (15.3%). At the sub-criterion level, clinical expert involvement (0.132), privacy policy availability (0.118), and guideline compliance (0.115) emerged as the most important determinants of application quality. These findings indicate that experts prioritize evidence-based healthcare support, professional oversight, and responsible data management when evaluating digital mental health applications.

The TOPSIS analysis successfully differentiated application performance and identified A1, A2, and A3 as the highest-performing applications, with closeness coefficients of 0.842, 0.816, and 0.791, respectively. In contrast, A10 achieved the lowest coefficient among the evaluated applications (0.581). The ranking results suggest that applications demonstrating balanced performance across clinical, ethical, and usability dimensions are more likely to achieve superior overall quality.

The sensitivity analysis further confirmed the robustness of the proposed framework. High-ranking stability was observed across alternative weighting scenarios, and Spearman correlation coefficients exceeding 0.90 indicated strong agreement between baseline and modified rankings. These findings demonstrate that the evaluation outcomes remain reliable despite moderate variations in decision-maker preferences.

This study contributes to the digital mental health evaluation literature by extending existing assessment frameworks through the integration of uncertainty-aware weighting and multi-criteria ranking within a unified analytical framework. Unlike conventional checklist-based evaluation methods, the proposed hybrid Fuzzy AHP–TOPSIS approach enables transparent prioritization of evaluation criteria and objective comparison of competing applications. The framework therefore provides a practical decision support tool for healthcare professionals, developers, researchers, and policymakers seeking to identify safe, clinically credible, and user-oriented digital mental health applications.

## Data Availability

Publicly available data were analyzed in this study. The Mobile Health Apps Evaluation Dataset is available through the Kaggle repository at: https://www.kaggle.com/datasets/subhamsarkar2002/mobile-health-apps-evaluation-dataset/data. The expert questionnaire developed for the Fuzzy Analytic Hierarchy Process (Fuzzy AHP), the linguistic scale for pairwise comparisons, and all supplementary methodological materials, including the evaluation framework and supporting documentation, are provided as Supplementary materials accompanying this article. Additional information related to the study is available from the corresponding author upon reasonable request.
