# Supplementary material for: A user centered evaluation framework for mobile health applications using Fuzzy AHP and TOPSIS: evidence from expert reviews and user experience data
Source: Front Public Health. 2026 Aug 14;14:1907690. doi: 10.3389/fpubh.2026.1907690 (PMC13522130; doi:10.3389/fpubh.2026.1907690)
Supplement: Supplementary file 1 [file Table_1.DOCX]

**Expert Questionnaire for Fuzzy AHP Analysis**

Instructions

Dear Expert,

This questionnaire aims to determine the relative importance of the criteria used to evaluate mobile health applications. Please compare each pair of criteria based on their importance in assessing the quality of mobile health applications.

Please use the following linguistic scale.

| Linguistic Term | Triangular Fuzzy Number |
| --- | --- |
| Equally Important | (1, 1, 1) |
| Slightly More Important | (1, 2, 3) |
| Moderately More Important | (2, 3, 4) |
| Strongly More Important | (4, 5, 6) |
| Very Strongly More Important | (6, 7, 8) |
| Extremely More Important | (8, 9, 10) |

**Part A. Expert Profile**

1. Gender
   - Male
   - Female
   - Prefer not to say
2. Age Group
   - 25–34
   - 35–44
   - 45–54
   - 55+
3. Province/Region
4. Professional Background
   - Public Health
   - Clinical Practice
   - Digital Health
   - Health Informatics
   - Mobile Application Development
   - User Experience Design
   - Other
5. Years of Professional Experience
   - <5 years
   - 5–10 years
   - 11–15 years
   - 15 years

**Part B. Pairwise Comparison of Main Criteria**

Goal: Evaluate Mobile Health Applications

Please indicate which criterion is more important.

**B1. Main Criteria Comparison Matrix**

| Comparison | Preference |
| --- | --- |
| Usability vs Engagement | ____ |
| Usability vs Clinical Relevance | ____ |
| Usability vs Ethics and Privacy | ____ |
| Engagement vs Clinical Relevance | ____ |
| Engagement vs Ethics and Privacy | ____ |
| Clinical Relevance vs Ethics and Privacy | ____ |

**Part C. Pairwise Comparison of Usability Subcriteria**

Criterion: Usability

Subcriteria:

U1 Ease of Use

U2 Learnability

U3 User Confidence

| Comparison | Preference |
| --- | --- |
| U1 vs U2 | ____ |
| U1 vs U3 | ____ |
| U2 vs U3 | ____ |

**Part D. Pairwise Comparison of Engagement Subcriteria**

Criterion: Engagement

Subcriteria:

E1 Interaction Quality

E2 Intention to Use

E3 User Engagement Mechanisms

| Comparison | Preference |
| --- | --- |
| E1 vs E2 | ____ |
| E1 vs E3 | ____ |
| E2 vs E3 | ____ |

**Part E. Pairwise Comparison of Clinical Relevance Subcriteria**

Criterion: Clinical Relevance

Subcriteria:

C1 Clinical Expert Involvement

C2 Guideline Compliance

C3 Healthcare Integration

| Comparison | Preference |
| --- | --- |
| C1 vs C2 | ____ |
| C1 vs C3 | ____ |
| C2 vs C3 | ____ |

**Part F. Pairwise Comparison of Ethics and Privacy Subcriteria**

Criterion: Ethics and Privacy

Subcriteria:

P1 Privacy Policy Availability

P2 Data Sharing Transparency

P3 Safety Alerts

| Comparison | Preference |
| --- | --- |
| P1 vs P2 | ____ |
| P1 vs P3 | ____ |
| P2 vs P3 | ____ |

Thank you for your participation. Your responses will be used solely for academic research purposes.
